# Health-related quality of life and functional ability in patients with early arthritis during remission steered treatment: results of the IMPROVED study

**DOI:** 10.1186/ar4361

**Published:** 2013-10-31

**Authors:** Lotte Heimans, Kirsten VC Wevers-de Boer, KK Michel Koudijs, Karen Visser, Yvonne P Goekoop-Ruiterman, Joop B Harbers, Gerda M Steup-Beekman, Leroy R Lard, Bernard AM Grillet, Tom WJ Huizinga, Cornelia F Allaart

**Affiliations:** 1 Department of Rheumatology, Leiden University Medical Center, PO Box 9600, 2300 RC, Leiden, The Netherlands; 2 Department of Rheumatology, Haga Hospital, Leijweg, 275, 2545 CH, The Hague, The Netherlands; 3 Department of Rheumatology, Franciscus Hospital, PO Box 999, 4700 AZ, Roosendaal, The Netherlands; 4 Department of Rheumatology, Bronovo Hospital, Bronovolaan, 5, 2597 AX, The Hague, The Netherlands; 5 Department of Rheumatology, Haaglanden Medical Center, PO Box 432, 2501 CK, The Hague, The Netherlands; 6 Department of Rheumatology, ZorgSaam Hospital, Wielingenlaan, 2, 4535 PA, Terneuzen, The Netherlands

## Abstract

**Introduction:**

The aim of this study was to investigate patient reported outcomes (PROs) of functional ability and health related quality of life (HRQoL) in patients with early (rheumatoid) arthritis during one year of remission steered treatment.

**Methods:**

In this study, 610 patients with early rheumatoid arthritis (RA) or undifferentiated arthritis (UA) were treated with methotrexate (MTX) and tapered high dose of prednisone. Patients in early remission (Disease Activity Score (DAS) <1.6 after 4 months) tapered prednisone to zero and when in persistent remission, also tapered MTX. Patients not in early remission were randomized to either MTX + hydroxychloroquine + sulphasalazine + prednisone (arm 1) or to MTX + adalimumab (arm 2). Every 4 months, patients filled out the Health Assessment Questionnaire (HAQ) and the McMaster Toronto Arthritis Patient Preference Questionnaire (MACTAR), the Short Form 36 (SF-36) and visual analogue scales (VAS). Change scores were compared between treatment groups. The association with achieving remission was analyzed using linear mixed models.

**Results:**

During year 1, patients who achieved early remission had the most improvement in PROs with scores comparable to the general population. Patients in the randomization arms showed less improvement. Scores were comparable between the arms. There was a significant association between achieving remission and scores of HAQ, MACTAR and physical HRQoL.

**Conclusions:**

In early arthritis, PROs of functional ability and HRQoL after one year of remission steered treatment reach normal values in patients who achieved early remission. In patients not in early remission, who were randomized to two strategy arms, PROs improved less, with similar scores in both treatment arms.

**Trial registrations:**

ISRCTN11916566 and EudraCT2006-006186-16

## Introduction

In rheumatoid arthritis (RA), treatment with disease-modifying antirheumatic drugs (DMARDs) is targeted at achieving optimal suppression of disease activity. With that treatment, clinical symptoms as well as radiological joint damage (progression) are prevented and patient-reported outcomes (PROs) such as pain, health-related quality of life (HRQoL) and physical and mental well-being improve [1]. Earlier studies have suggested that the better disease activity is suppressed, the better the outcomes regarding function and radiological joint damage progression [2,3]. Achieving clinical remission would ideally be associated with achieving PROs comparable to those in the general population.

In the Induction therapy with Methotrexate and Prednisone in Rheumatoid or Very Early arthritic Disease (IMPROVED) study, DMARD treatment was targeted at achieving remission. Patients with early RA were initially treated with combination therapy comprising methotrexate (MTX) and prednisone. If clinical remission (Disease Activity Score (DAS) <1.6) was not achieved within 4 months, patients were randomized into two treatment arms: either with a combination of nonbiologic DMARDs and low-dose prednisone or with MTX and the TNF-α inhibitor adalimumab (ADA). The aim of this subanalysis was to measure change in functional ability and HRQoL during the first year of remission-steered treatment to compare outcomes between the randomization arms and to compare study patients with the general population.

## Methods

### Study design

The IMPROVED study is a multicenter, randomized, single-blind trial comparing two combination therapies in patients with recent-onset arthritis. Its aim is achieving clinical remission, which is defined as a DAS less than 1.6. The IMPROVED trial was designed and conducted by rheumatologists in the Foundation for Applied Rheumatology Research and is registered in the International Standard Randomised Controlled Trial Register (ISRCTN11916566) and the European Clinical Trials Database (EudraCT2006-006186-16).

Patients were recruited between March 2007 and September 2010 from 12 hospitals in the western part of The Netherlands. The medical ethics committee of each participating center approved the study protocol (see Acknowledgements), and all patients gave their written informed consent to participate. Patients with RA and patients with undifferentiated arthritis (UA) were included. RA was diagnosed according to the 2010 American College of Rheumatology and European League against Rheumatism (ACR/EULAR) classification criteria [4] with duration of symptoms less than 2 years. UA was defined as arthritis in at least one joint and one other painful joint in which no definitive diagnosis could be made, which was considered to be early RA according to the treating rheumatologist, regardless of symptom duration. All patients were at least 18 years old and had a DAS of 1.6 or higher. Detailed inclusion and exclusion criteria have been described previously [5].

All patients were initially treated for 4 months with MTX 25 mg/wk and a tapered high dose of prednisone, starting with 60 mg/day and tapered to 7.5 mg/day during the course of 7 weeks. For patients in early remission (DAS less than 1.6 after 4 months), prednisone was tapered to 0, and, if still in remission after 8 months, MTX was also tapered to 0. Patients not in early remission (DAS 1.6 or higher) were randomized using variable block randomization and stratified per center to ensure numerical equality of the two treatment groups. The randomization sequence was obtained by computer. At the local centers, allocation of UA and RA patients was performed by drawing opaque envelopes from separate boxes. Patients were randomized to either a combination of either (1) MTX 25 mg/wk, hydroxychloroquine (HCQ) 400 mg/day, sulfasalazine (SSZ) 2,000 mg/day and prednisone 7.5 mg/day (arm 1) or (2) a combination of ADA 40 mg/2 weeks and MTX 25 mg/wk (arm 2). When patients did not achieve remission within 8 months, those in arm 1 were switched to ADA + MTX and for those in arm 2, the dosage of ADA was increased to 40 mg/wk. For patients in both arms who achieved remission within 8 months, treatment was tapered to MTX monotherapy. Patients who did not achieve remission but were not randomized were analyzed as a separate group, called the outside protocol (OP) subgroup [6].

### Outcomes

Functional ability was assessed every 4 months with the Health Assessment Questionnaire (HAQ) [7]. The HAQ score of the general Finnish population is 0.25 [8].

The McMaster Toronto Arthritis Patient Preference Questionnaire (MACTAR) also measures functional ability. Patients rank five activities in which they are impaired because of their arthritis. Over time, improvement or deterioration of these five activities can be measured. The MACTAR is sensitive to change and useful for the detection of small differences. Compared to the baseline score, a higher score denotes improvement and a lower score indicates deterioration. The MACTAR interview from Canada was translated into Dutch in collaboration with the author of the original MACTAR. The translation was first used in the COBRA study of combination therapy in rheumatoid arthritis, in which it was validated and adjudged to be highly responsive [9-11].

HRQoL was assessed using the 36-Item Short Form Health Survey (SF-36), focusing on eight domains of health: physical functioning, role limitations due to physical or emotional functioning, bodily pain, general health, vitality, social functioning and mental health. The total score ranges from 0 (worst) to 100 (best). Two summary component scores, the Mental Component Score (MCS) and the Physical Component Score (PCS), can be calculated from among the eight domains. These component scores are standardized on the basis of worldwide population norms to a mean of 50 and a standard deviation of 10 [12,13]. The minimum clinically important difference for assessing improvement or deterioration is a 5- to 10-point difference from baseline for the subscales and from 2.5 to 5 points for the component scores [14].

Various Visual Analogue Scales (VASs) were used, on which patients had to indicate on a scale from 0 to 100 mm (0 means none and 100 means the worst) their rating of their global health (VAS_gl_), pain (VAS_pain_), disease activity (VAS_da_) and morning stiffness (VAS_ms_).

### Statistical analyses

All outcomes were calculated according to the intention-to-treat principle. All mean outcomes after 4 months, 8 months and 1 year were tested between arms 1 and 2 using Student’s *t-*test, and we used the χ^2^ test to test the difference in remission rates.

HAQ, MACTAR, MCS, PCS and VAS scores were reported separately for patients who achieved early remission and those who were randomized, and the scores were compared between the randomization arms. The results of the study population were compared with those in the general population, if those data were available.

Mean score changes over time were tested between the randomization arms using an independent Student’s *t*-test. Clinically relevant improvement or deterioration in HRQoL after 1 year was assessed per treatment group on the basis of the minimum clinically important difference.

To assess the relationship between achieving remission and the PROs PCS, MCS, HAQ and MACTAR, a linear mixed model (with an unstructured covariance scheme) was used. The analyses were first performed with an interaction term for remission achievement and treatment (early remission, arm 1, arm 2 and OP group) because the different treatment strategies might influence remission achievement.

As fixed effects were entered into the model: time (study visits at 4 months, 8 months and 1 year) and mean baseline score of the assessed PRO. In cases of a significant interaction term, the analyses were stratified for treatment. The association between remission and PROs was assessed with and without adjustment for the baseline variables anticitrullinated protein antibody (ACPA) status (positive or negative), sex (male or female), DAS at baseline, tender joint count and swollen joint count which were entered in the model as fixed effects. We used these determinants because they were identified as predictors for achieving remission after the first 4 months of the study [5]. After the initial analysis in which remission was defined as a DAS less than 1.6, we reanalyzed the association with remission defined according to the provisional Boolean-based remission definition published by ACR/EULAR with a 44-joint count DAS [15]. Statistical analyses were conducted using SPSS for Windows version 20.0 software (SPSS, Inc, Chicago, IL, USA).

## Results

In total, 610 patients were included. During the first year, 32 patients left the trial (23 withdrew consent, 3 discontinued because of a revised diagnosis and 6 dropped out because of comorbidity). After 4 months, 387 achieved early remission (DAS less than 1.6). Of the 221 patients who did not achieve early remission, 161 patients were randomized: 83 patients into arm 1 (poly-DMARDs) and 78 into arm 2 (ADA + MTX). Fifty patients did not achieve remission but were not randomized (assigned to the OP subgroup) [6]. Patients who achieved early remission had lower mean baseline DAS, lower values of all DAS components and shorter symptom duration and included fewer females and more patients positive for ACPA (Table 1) [5].

**Table 1:** **Baseline characteristics of all patients**^**a**^

	**Early remission**	**Arm 1**	**Arm 2**	**OP group**
**Baseline characteristics**	**(*N*** **= 387)**	**(*N*** **= 83)**	**(*N*** **= 78)**	**(*N*** **= 50)**
Mean age (±SD), years	52 ± 14	48 ± 14	51 ± 14	54 ± 14
Females, *n* (%)	239 (62)	63 (76)	64 (82)	42 (84)
Symptom duration, weeks	17 (9 to 30)	22 (9 to 40)	21 (8 to 29)	18 (9 to 42)
ACPA-positive, *n* (%)	225 (58)	40 (48)	36 (46)	25 (50)
RA2010, *n* (%)	297 (77)	66 (80)	64 (82)	40 (80)
Erosive disease, *n* (%)	63 (16)	10 (12)	13 (17)	3 (6)
DAS, mean ± SD	3.0 ± 0.9	3.6 ± 0.9	3.6 ± 1.0	3.6 ± 0.9
Tender joint count, median (IQR)	5 (2 to 9)	6 (3 to 10)	8 (4 to 12)	7 (3 to 13)
Swollen joint count, median (IQR)	5 (3 to 8)	8 (6 to 13)	9 (6 to 13)	8 (6 to 14)
HAQ, mean ± SD	1.0 ± 0.7	1.4 ± 0.6	1.4 ± 0.65	1.3 ± 0.7
MCS, mean ± SD	51.2 ± 10.2	46.1 ± 12.4	48.8 ± 11.5	46.5 ± 13.3
PCS, mean ± SD	37.6 ± 9.3	33.0 ± 8.8	32.9 ± 8.9	35.2 ± 8.5
Mean MACTAR, mean ± SD	50.1 ± 4.5	47.7 ± 4.6	48.1 ± 4.6	47.7 ± 5.2
Mean VAS_gl_ (±SD), mm	43 ± 24	54 ± 20	54 ± 22	51 ± 22
Mean VAS_da_ activity (±SD), mm	56 ± 25	66 ± 19	67 ± 22	66 ± 20
Mean VAS_pain_ (±SD), mm	50 ± 24	63 ± 19	61 ± 20	60 ± 24
Mean VAS_ms_ (±SD), mm	56 ± 27	69 ± 21	62 ± 25	54 ± 30

^a^Data are presented as mean ± SD, median (IQR or number (%). ACPA: anticitrullinated protein antibody; DAS: Disease Activity Score; HAQ: Health Assessment Questionnaire; MACTAR: McMaster Toronto Arthritis Patient Preference Questionnaire; MCS: Mental Component Score on the 36-Item Short Form Health Survey; OP group: outside protocol subgroup; PSC: Physical Component Score on the 36-Item Short Form Health Survey; RA2010: rheumatoid arthritis according to the 2010 American College of Rheumatology and European League against Rheumatism classification criteria [4]; VAS_da_: disease activity; VAS_gl_: global health; VAS_ms_: morning stiffness; VAS_pain_: pain.

At the 1-year follow-up visit, we found that remission was most often achieved by patients in the early remission group (68%). Fewer patients randomized to arm 1 had achieved remission at 1 year than patients randomized to arm 2 (25% and 40%, respectively; *P* = 0.01) (Table 2).

**Table 2:** **Patient-related outcomes of all patients during 1 year of follow-up**^**a**^

	**Early remission**	**Arm 1**	**Arm 2**		**OP group**
**Patient characteristics at follow-up visits**	**(*N*** **= 387)**	**(*N*** **= 83)**	**(*N*** **= 78)**	***P***	**(*N*** **= 50)**
4 months					
DAS	0.97 (0.40)	2.49 (0.63)	2.57 (0.68)	0.47	2.31 (0.63)
HAQ	0.23 (0.33)	0.86 (0.57)	0.88 (0.57)	0.77	0.73 (0.68)
MACTAR	58.2 (15.7)	52.8 (15.1)	48.9 (18.8)	0.14	51.6 (14.1)
MCS	52.4 (8.0)	48.8 (9.9)	50.7 (10.8)	0.26	49.8 (10.5)
PCS	51.7 (8.1)	39.4 (9.7)	38.1 (9.4)	0.44	42.5 (9.4)
VAS_gl_, mm	14 (14)	37 (21)	39 (21)	0.61	28 (22)
VAS_da_, mm	12 (15)	42 (24)	43 (24)	0.74	32 (25)
VAS_pain_, mm	10 (14)	39 (24)	38 (24)	0.79	27 (24)
VAS_ms_, mm	11 (17)	40 (27)	39 (27)	0.78	32 (30)
8 months					
DAS	1.29 (0.69)	1.97 (0.87)	2.01 (0.91)	0.77	2.02 (0.84)
HAQ	0.35 (0.44)	0.74 (0.61)	0.81 (0.64)	0.51	0.68 (0.59)
MACTAR	56.4 (15.7)	55.8 (14.7)	54.5 (16.1)	0.60	48.9 (19.9)
MCS	52.9 (8.4)	46.6 (17.9)	48.7 (10.3)	0.85	48.5 (13.0)
PCS	48.9 (9.1)	42.8 (10.9)	42.5 (11.0)	0.26	43.7 (9.5)
VAS_gl_, mm	20 (20)	33 (23)	34 (21)	0.75	30 (23)
VAS_da_, mm	22 (23)	39 (26)	33 (24)	0.20	35 (25)
VAS_pain_, mm	19 (23)	35 (26)	31 (25)	0.36	32 (24)
VAS_ms_, mm	24 (26)	34 (29)	37 (28)	0.51	40 (27)
1 year					
DAS	1.31 (0.78)	2.07 (0.89)	1.77 (0.90)	0.04	2.20 (0.83)
HAQ	0.38 (0.49)	0.87 (0.66)	0.81 (0.66)	0.60	0.77 (0.65)
MACTAR	63.0 (9.4)	59.2 (10.3)	60.4 (11.9)	0.54	59.7 (11.21)
MCS	53.1 (8.6)	50.5 (10.3)	50.5 (10.1)	0.97	50.4 (11.9)
PCS	48.6 (9.8)	39.9 (10.3)	43.0 (11.4)	0.10	42.6 (10.9)
VAS_gl_, mm	20 (21)	33 (23)	27 (20)	0.10	33 (24)
VAS_da_, mm	24 (26)	42 (29)	31 (26)	0.02	34 (27)
VAS_pain_, mm	21 (23)	38 (28)	28 (25)	0.02	28 (25)
VAS_ms_, mm	25 (26)	41 (31)	33 (27)	0.96	39 (30)
Remission (DAS <1.6)	263 (68)	21 (25)	32 (41)	0.01	11 (22)

^a^Data are means ± SD, medians (IQR) or numbers (%) as appropriate. ACPA: anticitrullinated protein antibody; DAS: Disease Activity Score; HAQ: Health Assessment Questionnaire; MACTAR: McMaster Toronto Arthritis Patient Preference Questionnaire; MCS: Mental Component Score on the 36-Item Short Form Health Survey; PSC: Physical Component Score on the 36-Item Short Form Health Survey; RA2010: rheumatoid arthritis according to the 2010 American College of Rheumatology and European League against Rheumatism classification criteria; VAS_da_: disease activity; VAS_gl_: global health; VAS_ms_: morning stiffness; VAS_pain_: pain. *P*-value represents the difference in mean scores and remission rates between arms 1 and 2.

### Functional ability

**Figure 1:**
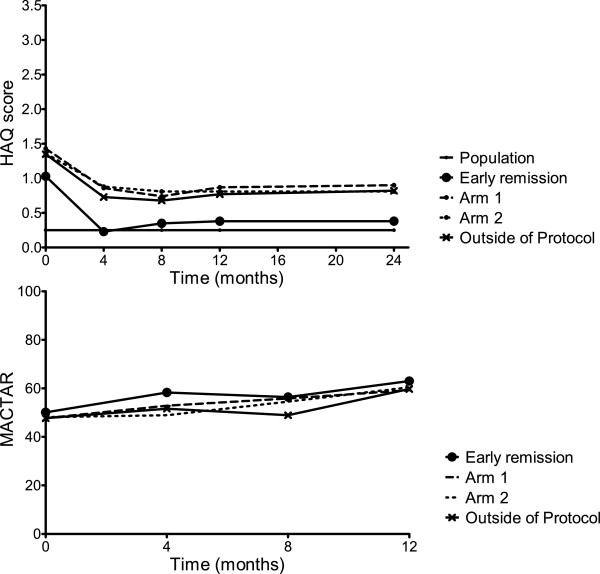
**Functional ability as measured by the Health Assessment Questionnaire and the McMaster Toronto Arthritis Patient Preference Questionnaire.** Scores in the first year in the general population (only for Health Assessment Questionnaire (HAQ)), the early remission group, arm 1, arm 2 and the outside protocol subgroup. MACTAR: McMaster Toronto Arthritis Patient Preference Questionnaire.

HAQ scores in the early remission group were lower, indicating better functional ability, than in the randomization arms, both at baseline and after 1 year (Figure 1). Functional ability improved the most during the first 4 months in all patients (Figure 1). The mean improvement in HAQ during the first year was comparable between arms 1 and 2 (mean difference = −0.005, 95% CI = −0.3 to 0.2). In the early remission group, the mean HAQ score after 1 year, 0.38, was closest to the general population mean of 0.25 (compared to mean HAQ scores of 0.87 in arm 1 and 0.88 in arm 2) (Figure 1 and Table 2).

Functional ability measured by the MACTAR, which is more sensitive to change than the HAQ, improved in all groups, together with continuous improvements in mean DAS (Tables 1 and 2). The mean change in MACTAR in year 1 was not significantly different between arms 1 and 2 (mean difference = −1.1, 95% CI = −5.2 to 3.1). The outcomes of the OP group were comparable to those in arms 1 and 2.

### Health-related quality of life

**Figure 2:**
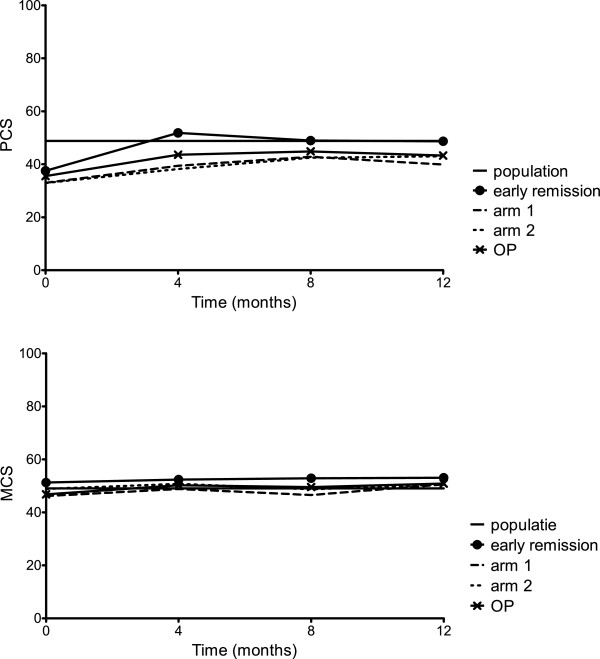
**Summary components scores of health as measured by the 36-Item Short Form Health Survey.** The 36-Item Short Form Health Survey (SF-36) Mental Component Score (MCS) and Physical Component Score (PCS) can be calculated from the eight SF-36 domains (physical functioning, role limitations due to physical functioning, bodily pain, general health, vitality, social functioning, role limitations due to emotional functioning and mental health) [12,13]. OP: outside protocol subgroup.

**Figure 3:**
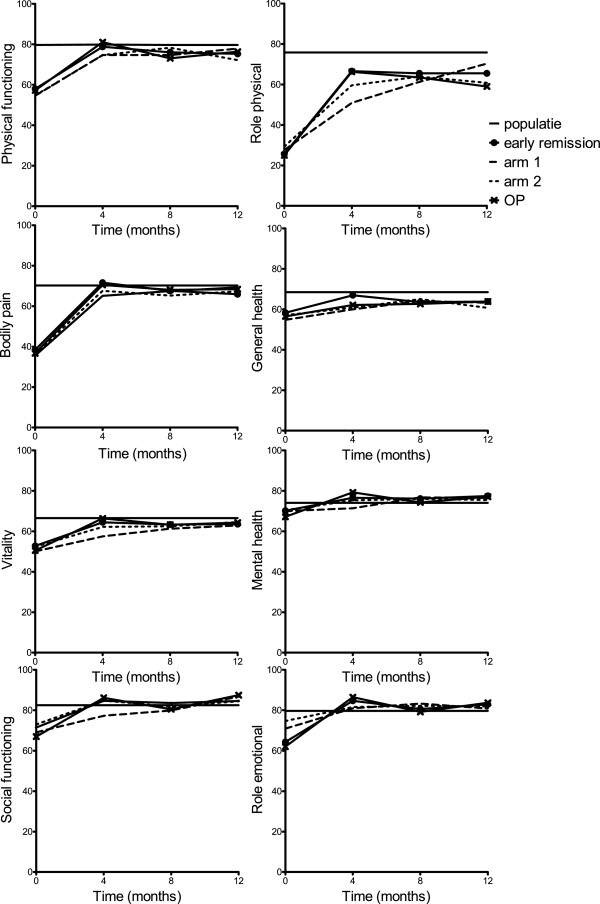
**The eight domains of health measured by the 36-Item Short Form Health Survey.** For the eight domains measured by the 36-Item Short Form Health Survey (physical functioning, role limitations due to physical functioning, bodily pain, general health, vitality, social functioning, role limitations due to emotional functioning and mental health), the total score ranges from 0 (worst) to 100 (best). OP: outside protocol subgroup.

At baseline in all groups, mental HRQoL measured with the MCS was higher than physical HRQoL measured with the PCS (Table 1 and Figure 2). Overall, the MCS at baseline was already close to the population average of 50, and improvement during the first year was minimal (Table 1 and Figure 2), although clinically relevant in the randomization arms based on the minimal clinically important difference in mean component scores of 2.5 to 5 points (improvement in arm 1 = 3.8 ±11.4, improvement in arm 2 = 2.8 ± 10.0). The mean improvement after 1 year was not significantly different between arms 1 and 2 (mean difference = 1.0, 95% CI = −2.8 to 4.7). The domains in which the most improvement was seen were the roles emotional and social functioning (Figure 3).

For the PCS, baseline scores in all groups were below the population average of 50 (Table 1 and Figure 2). The early remission group improved to the population average during the first 4 months of treatment and stabilized, whereas the patients in the randomization arms also improved during the first 4 months and stabilized, but below the population average (Table 2 and Figure 2). The mean improvement (±SD) at 1 year was clinically relevant in all groups based on the minimal clinically important difference of 2.5 to 5 points in the early remission group, 11.1 (SD 11.7); arm 1, 8.0 (10.9), and arm 2, 10.1 (12.8). The mean improvement at 1 year between patients who did versus those who did not achieve early remission was significantly higher in patients who achieved early remission (mean difference = −2.7, 95% CI = −4.9 to 0.5). There was no significant difference between arms 1 and 2 (mean difference = −2.1, 95% CI = −6.3 to 2.1). The domains in which most improvement was seen were physical functioning, bodily pain and role limitations due to physical functioning (Figure 3). Again, MCS and PCS in the OP group were comparable to those scores in arms 1 and 2.

### Visual Analogue Scale scores

Patients who achieved early remission had lower VAS scores (indicating better outcomes) at baseline and at 1 year than patients in the randomization arms (Tables 1 and 2). Patients in arm 2 reported lower VAS scores than patients in arm 1 at 1 year (Table 2). Only the VASda showed more improvement at 1 year in arm 2 than in arm 1 (mean difference = 13, 95% CI = 2 to 23). For the other VAS scores, the improvements were comparable between the randomization arms (mean difference (95% CI) VAS_gl_ = 7 (−2 to 16), VAS_pain_ = 9 (−1 to 19) and VAS_ms_ = 7 (5 to 16). The OP group had results similar to those of patients in arms 1 and 2.

### Association of patient-reported outcomes with remission

The analyses of the HAQ score and the PCS were stratified by treatment group because there was an interaction between treatment group and achieving remission, with remission defined as DAS less than 1.6. The association between HAQ and achieving remission and between PCS and achieving remission was significant in all groups during the first year of the study (Table 3). The analyses for MACTAR and MCS were not stratified. In the total study group, there was a significant association between MACTAR and achieving remission. There was also a significant association between MCS and achieving remission in the total study group, but after adjustment (for ACPA status (positive or negative), sex (male or female), DAS at baseline and tender and swollen joint counts at baseline), this association was no longer found (Table 3). Results were the same when we used the ACR/EULAR provisional remission definition (data not shown).

**Table 3:** **Association between patient-reported outcomes and remission during 1 year of follow-up**^**a**^

**Measurements**	**All**	**Early remission**	**Arm 1**	**Arm 2**	**OP group**
Crude β (95% CI)					
HAQ	–	−0.31 (−0.36 to −0.26)	−0.43 (−0.57 to −29)	−0.45 (−0.58 to −0.32)	0.18 (−0.33 to −0.02)
MACTAR	7.8 (6.9 to 8.9)	–	–	–	–
PCS	–	6.2 (5.1 to 7.4)	10.2 (7.5 to 12.9)	8.9 (5.8 to 12.0)	4.5 (0.6 to 8.4)
MCS	0.8 (0.01 to 1.6)	–	–	–	–
Adjusted β (95% CI)					
HAQ	–	−0.30 (−0.35 to −0.25)	−0.43 (−0.57 to −29)	−0.45 (−0.58 to −0.32)	0.17 (−0.32 to −0.01)
MACTAR	8.1 (7.0 to 9.2)	–	–	–	–
PCS	–	6.0 (4.9 to 7.2)	9.9 (7.1 to 12.7)	9.1 (6.1 to 12.1)	4.2 (0.2 to 8.1)
MCS	0.8 (−0.01 to 1.7)	–	–	–	–

^a^CI: confidence interval; HAQ: Health Assessment Questionnaire; MACTAR: McMaster Toronto Arthritis Patient Preference Questionnaire; MCS: Mental Component Score of the 36-Item Short Form Health Survey; OP: outside protocol subgroup; PCS: Physical Component Score of the 36-Item Short Form Health Survey. Fixed effects were entered for time (study visits at 4 months, 8 months and 1 year) and mean baseline score of the assessed PRO. The analyses were also performed with adjustment (adjusted β) for ACPA status (positive or negative), sex (male or female), DAS at baseline and tender and swollen joint counts, and these were also entered into the analysis as fixed variables. For HAQ score and PCS, we stratified for treatment group (early remission, arm 1, arm 2 and OP subgroup) because there was a significant interaction between treatment group and achieving remission.

## Discussion

We assessed PROs of functional ability and HRQoL in patients with UA and early RA who were treated with the aim of achieving remission (DAS less than 1.6). Patients who had achieved early remission at 4 months had the best PROs at baseline and during the first year of the study, and only in these patients did PROs reach levels comparable to those measured in the general population. Patients who did not achieve early remission and were randomized to therapy with multiple DMARDs with prednisone or a combination of MTX with ADA had lower, and between arms comparable, PRO scores during the first year.

At baseline, the IMPROVED study population, which had a mean age of 52 years, scored lower on all domains of the physical HRQoL compared to healthy individuals in the Dutch population older than 70 years of age [12]. Therefore, it seems that the disease burden of early arthritis is substantial. With treatment, the component score for physical HRQoL showed a clinically relevant improvement in all groups, with the most improvement occurring in the early remission group during the first 4 months. The mental HRQoL remained stable around the population average during the first year of treatment, which suggests that the impact of early arthritis is mainly physical. This was also shown in previous published studies [1,16]. However, improvement of physical HRQoL and HAQ scores to the population average in the first year after diagnosis in a remission-steered treatment protocol was not reported earlier [1,17].

It is generally accepted that remission is the optimal treatment target in RA. Ideally, remission would result in patients having no radiological joint damage progression, no symptoms and no limitations; in other words, normality, functional ability and quality of life comparable to that of the general population. More than DASs, PROs show whether such improvement can be achieved if treatment is steered at achieving remission. The current results indicate that scores comparable to those in the general population can indeed be achieved, but mainly in patients who were in early remission after 4 months of initial treatment. There might be a two-sided relationship between early remission and better PRO scores, because patients who achieved early remission had better PRO scores at baseline than patients who did not. This indicates that a predisposition to achieving remission determines outcomes. Our results indicate that patients with milder disease or better predisposition to achieving remission benefit from remission-steered treatment because this allows them to achieve normal levels of functional ability and quality of life, which may have a significant impact on their ability to work and decrease the personal and societal costs associated with RA [18,19]. The magnitude of the association between remission and the various PROs was actually bigger in arms 1 and 2 than in the early remission group, which had better PROs after 1 year as well as better PROs at baseline than the patients in arms 1 and 2. This suggests that, regardless of baseline score, achieving remission itself is associated with PRO improvement.

One may also argue that, without treatment, the arthritis in these patients would have regressed and function and quality of life would have been restored. However, we previously showed that a majority of patients who achieved remission were ACPA-positive, which makes spontaneous remission less likely [5].

Although significantly more patients in arm 2 than in arm 1 had achieved remission at 1 year, we found no significant differences in improvement of functional ability, HRQoL and VAS results between arms. Only VAS_da_ as rated by the patient improved more in arm 2 than in arm 1. Despite continued treatment adjustments targeted at remission, remission percentages in both arms remained lower than in the early remission group. Possibly as a consequence, functional ability and HRQoL in the physical domain did not reach the same levels as in the early remission group. In particular, HAQ scores were higher in the randomization arms than in the early remission group and physical HRQoL did not reach the levels found in the general population. Although we found that PROs were associated with achieving remission and that significantly more patients in arm 2 than in arm 1 achieved remission at 1 year, we found no significant differences in improvement of functional ability and HRQoL between arms. Only improvement in VAS_da_ was significantly better in arm 2 patients than in arm 1 patients, which can be explained by significantly lower mean DASs in arm 2 and may also be related to higher patient expectations associated with earlier introduction of the subcutaneous TNF inhibitor ADA in this treatment arm [20,21]. Overall, disease activity was well-suppressed in both arms, which may explain why we found no differences in improvement in HAQ score and HRQoL. The actual DAS, rather than having a score just above or below the threshold of remission, may be the main determinant of PROs. The patients in the OP subgroup had results similar to those of patients in arms 1 and 2, which can be explained by the comparable response to initial treatment.

## Conclusion

In patients with early RA, there is an association between achieving remission and having better functional ability, HRQoL and other PROs, which may in part be bidirectional. The condition of patients who achieve early remission improves and remains at levels similar to those of the general population. This finding supports the idea that early remission-steered treatment could result in complete suppression of symptoms with normal functioning and may prevent chronic deterioration in PROs.

## Abbreviations

ACPA: Anticitrullinated protein antibody; ACR/EULAR: American College of Rheumatology/European League against Rheumatism; ADA: Adalimumab; CI: Confidence interval; DAS: Disease activity score; DMARD: Disease-modifying antirheumatic drug; HAQ: Health assessment questionnaire; HCQ: Hydroxychloroquine; HRQoL: Health-related quality of life; IMPROVED: Study Induction therapy with Methotrexate and Prednisone in Rheumatoid or Very Early arthritic Disease; MACTAR: McMaster Toronto Arthritis Patient Preference Questionnaire; MCS: Mental component score; MTX: Methotrexate; OP: Outside protocol; PCS: Physical component score; PRO: Patient-reported outcome; RA: Rheumatoid arthritis; SF-36: 36-Item Short Form Health Survey; TNF: Tumor necrosis factor; UA: Undifferentiated arthritis; VAS: Visual analogue scale; VASda: Visual analogue scale disease activity; VASgl: Visual analogue scale global health; VASms: Visual analogue scale morning stiffness; VASpain: Visual analogue scale pain.

## Competing interests

The authors have no competing interests to declare.

## Authors’ contributions

LH performed the statistical analyses, interpreted the data and drafted the manuscript. KB and KV contributed to the acquisition of data. MK contributed to the statistical analyses. YG, JH, GS, LL and BG participated in the study design and contributed to the acquisition of data. TH participated in the study design, contributed to the acquisition of data and revised the manuscript. CA participated in the study design and contributed to the acquisition of data, was involved in analyzing and interpreting the data and helped to draft the manuscript. All authors read and approved the final version of the manuscript.
